# Chemical Softness in Aromatic Adsorption: Benzene, Nitrobenzene and Anisole on Pt{111}

**DOI:** 10.1021/acs.jpca.4c02214

**Published:** 2024-07-22

**Authors:** Amy L. Gunton, Stephen J. Jenkins

**Affiliations:** Yusuf Hamied Department of Chemistry, University of Cambridge, Lensfield Road, Cambridge CB2 1EW, U.K.

## Abstract

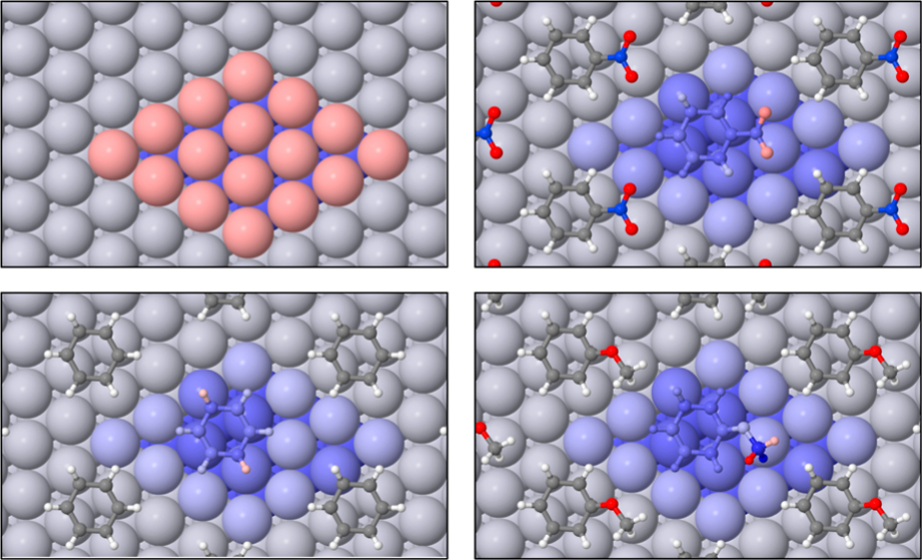

We describe a method for the calculation of chemical softness at metal surfaces, demonstrating its utility in understanding the adsorption of benzene, nitrobenzene and anisole at the Pt{111} surface. Based on this method, we show that directing effects due to either of the substituent groups are mostly swamped by substrate influences, while significant variations in softness within the groups themselves are readily apparent.

## 1 Introduction

One of the most challenging tasks for first-principles computational modeling of surface/molecule interactions is that of predicting how modification of electronic structure may result in selective reactivity. With high-quality methods such as density functional theory (DFT), it is possible, of course, to identify transition states for different competitive surface reactions, ordering them according to their computed activation energies, and subsequent examination of calculated orbitals may yield insight into the reasons why one may be preferred over all others. But such an approach is demanding upon resources, and rather specific to the details of a given situation. How much more attractive, therefore, to have access to some form of reactivity index that somehow predicts the likelihood of chemical processes without explicitly computing transition states but maintaining sufficient accuracy to model trends. Such an index would permit, for example, high-throughput screening of potential catalysts, identifying promising candidates for more detailed further investigation by more traditional means.

Undoubtedly the most widespread and successful reactivity index in the context of surface science is the d-band center concept, whereby the proximity of the eponymous band’s center-of-gravity to the Fermi level of the bare substrate is taken as a proxy for reactivity.^1−3^ This approach typically informs descriptions of the relative activity of different metals and their alloys toward the dissociation of molecules on arrival at the surface or shortly thereafter. What it lacks, however, is any reference to the locality of chemical reactivity, in the sense of which part of a surface may be most reactive, or which part of a molecule may be most likely to react.

Alternative approaches, such as the Wilke function^4^ and the local electron attachment energy,^5^ have been proposed as local reactivity indices for use at metal surfaces, but have not yet gained widespread traction as convenient analysis tools. In contrast, the concept of chemical softness,^6^ derived as an extension to the older concept of the Fukui function,^7−9^ has become quite well-known in the context of reactivity in isolated atoms or molecules. There are, unfortunately, some obstacles to its application at metal surfaces, which we address in this paper. To begin with, however, let us review the underlying equations, before introducing the physical systems that we shall take as our test cases.

### 1.1 Quantifying Chemical Softness

For a system containing *N* electrons, the global softness was defined by Berkowitz and Parr^6^ as
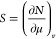
1where μ is the electronic chemical potential and *v* the external potential. Generalizing this concept, the local softness could then be defined as
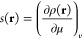
2where ρ(**r**) is the local electron density. Note that this local quantity simply integrates to yield the corresponding global quantity
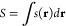
3as one might expect.

In order to evaluate the local softness in practice, therefore, it is necessary to raise or lower the chemical potential and observe the effect upon the local electron density. Crucially, however, this must be done while maintaining constant external potential. Clearly the application of a uniform external potential will raise or lower the absolute value of the chemical potential without materially altering the physical circumstances of the system. In contrast, raising the chemical potential while leaving the external potential unaltered must imply an increase in the total electron population of the system, just as lowering it under the same constraint would imply a decrease. For deeper context, a number of very pertinent observations have recently been made by Szarek concerning the relationship between chemical softness and the concepts of molecular self-capacitance and local density of states (DOS).^10^

Before considering in more detail the practicalities of actually calculating the local softness, it will be instructive to discuss how its interpretation at metallic surfaces relates to the aforementioned Fukui functions.^7−9^ These are respectively defined for nucleophilic (reduction) and electrophilic (oxidation) reactions as
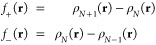
4where subscripts on the electron density terms now indicate the total number of electrons in the system. The mean of these functions
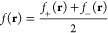
5then serves as a measure of radical reactivity. For a nonmetallic entity, the nucleophilic and electrophilic definitions are markedly distinct, the former involving changes in occupancy of the lowest unoccupied molecular orbital (LUMO) and the latter changes in occupancy of the highest occupied molecular orbital (HOMO). Since the HOMO and LUMO invariably differ from one another, so too do the corresponding Fukui functions.

In a metallic system, however, the two definitions of eq 4 probe changes in occupancy of states that lie only just above and just below the Fermi level, so that the nucleophilic and electrophilic Fukui functions both tend toward the radical Fukui function in the limit of an extended system. The local softness does not, therefore, require separate definitions for different directions of electron transfer - it describes oxidation, reduction, and isoelectronic processes at metallic surfaces with equanimity.

Turning now to practicalities, in the case of an isolated atom or molecule (described as such with boundary conditions set at infinity) the direct use of eq 2 poses little difficulty. Calculations can be carried out for charged instances of the system under study, and the resulting chemical potential can be unequivocally obtained with reference to the vacuum potential at infinite distance. The change in local electron density caused by the excess charge can then be divided by the corresponding change in chemical potential, and extrapolation down to the case of infinitesimal charge will yield the desired property. In contrast, a rather fundamental issue emerges when dealing with periodic systems of nonzero charge, since it is necessary in these to include a fictitious uniform potential within the unit cell in order to avoid certain infinities that would otherwise arise. In the case of an isolated atom or molecule described using periodic boundary conditions (as would be necessary within a plane-wave computer code) the vacuum potential of a charged system converges only rather slowly with the cell size, necessitating repeated calculations and extrapolation to obtain reliable results.^11^ For surfaces, as we shall see, the situation apropos convergence is rather more difficult.

One interesting approach to the calculation of chemical softness in extended systems, including at surfaces, has previously been applied to alkaline earth oxides.^12,13^ This relies upon an approximate reformulation of the softness as the limit of an integral over the local DOS
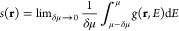
6which should be accurate so long as the relaxation of electronic states upon variation of charge is minimal. Such a condition may well be true for insulating systems but cannot confidently be assumed for metallic ones. Note also that the chosen approximation strictly corresponds to a definition of softness suitable for the description of electrophilic reactivity, which will be distinct from nucleophilic reactivity for insulating surfaces. The necessary modification to eq 6 for evaluation of nucleophilic softness is trivial but nevertheless underlines how the insulating surface case differs from our present concern.

To proceed further in the metallic surface case, however, it will instead be convenient to split the partial derivative of eq 2 using the chain rule, obtaining
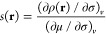
7where σ represents the surface charge density. The numerator here is straightforward to evaluate, requiring only the performance of charged calculations to gauge the resultant change in the local electron density. The difficulty associated with changing the chemical potential while keeping the external potential fixed (which necessitates access to a reliable reference energy) is confined solely to the denominator. We shall describe our approach to this difficulty in Calculation of the Denominator for the Clean Surface.

### 1.2 Aromatic Adsorption

The adsorption of aromatic molecules on metal surfaces has long been the subject of intense research activity, not least via the application of first-principles DFT.^14^ From the perspective of chemical softness, one of the most interesting questions is the extent to which the directing effects of various side groups remain important when chemisorbed at a catalytically active surface. For example, the electron-donating methoxy group in anisole is generally thought not only to enhance electrophilic attack generally, but also to direct it toward the *ortho* and *para* positions of the ring.^15^ The electron-withdrawing nitro group in nitrobenzene, on the other hand, ought to suppress electrophilic attack, but that which does occur should be directed toward the *meta* position. Do these rules of thumb hold when molecules of these species are chemisorbed, or does the interaction with the surface dominate?

This question has been partially addressed by one of us before, in relation to the adsorption of anisole on Pt{111}.^16^ In that work, the polarity of C–H bonds within the ring of the free molecule was found to be greatest in the *ortho* position, slightly less in the *para* position, and least in the *meta* position. When adsorbed on the surface, however, the polarity of all such C–H bonds is enhanced, but in a manner that is dominated by their positions with respect to surface bonding, not by their positions with respect to the methoxy group. In the present work, we wish to determine whether the chemical softness of adsorbed anisole more closely resembles the surface-dominant pattern or the molecule-dominant pattern.

For comparison, we also investigate nitrobenzene, to gauge how electron-withdrawing groups may or may not differ from electron-donating ones. We do not, it should be stressed, promote the present work as a comprehensive study of either substituted adsorbate, since we consider only a single adsorption site and orientation for each, chosen in light of the strong preference shown by unsubstituted benzene for one particular adsorption geometry. Instead, we benefit from this limitation in that similarities and differences between benzene, nitrobenzene and anisole may be more readily discerned if the fundamental geometry is constrained to one type across all three adsorbates.

## 2 Methodology

First-principles DFT calculations were carried out using the CASTEP computer code.^17^ Electronic wave functions were expanded in a basis set of plane waves, up to a kinetic energy cutoff at 340 eV, and exchange–correlation interactions were incorporated via the Perdew–Burke–Ernzerhof (PBE) functional.^18^ Electron–ion interactions were included through the use of ultrasoft pseudopotentials.^19^ For purposes of geometry optimization, the surface was modeled using a slab comprising four layers of platinum, in which the top two layers were allowed to relax under the influence of calculated forces. Adsorbates were placed on the relaxed side, at a coverage of 0.11 ML within a (3 × 3) surface unit cell. Brillouin-zone sampling was achieved on a 3 × 3 × 1 Monkhorst–Pack mesh.^20^ The same parameters were applied in calculating the numerator of eq 7 for the fully relaxed geometry thus obtained. In calculating the denominator (for details of which, see Calculation of the Denominator for the Clean Surface) we employed only a clean-surface model, described within a (1 × 1) surface unit cell using an 8 × 8 × 1 mesh for geometry optimization and a 30 × 30 × 1 mesh for DOS evaluation.^21^ Multiple different slab thicknesses and vacuum gaps were used, as we describe later. We expect this denominator to be dominated by the substrate rather than the adsorbate, and since it is a global property its precise value does not impact upon the local properties inherent within the numerator. When evaluating partial derivatives with respect to surface charge (for either numerator or denominator) we have taken finite differences between calculations performed at σ = ±0.02 e Å^–2^.

Two approaches were used to display softness values. In the first, the local softness was directly indicated by a colormap projected onto an isosurface of constant valence electron density (specifically, if arbitrarily, set to be one-fifth of the mean valence electron density for bulk platinum). This has the advantage that the softness is shown as a continuous variable, but clearly the visual impression would change somewhat if a different isosurface density value were to be chosen. In the second approach, we made use of Bader’s topological method to partition space into discrete regions associated with individual atoms within the molecule/surface system.^22^ Local softness was then integrated within each such topologically defined envelope, giving us a measure of atomic softness that we then displayed as a colormap for spherical atoms of arbitrarily chosen radii.

## 3 Results

As discussed above, we tackled the evaluation of local softness via eq 7, focusing first upon the denominator (a global property, which we calculate only for the clean surface) and then upon the numerator (a local property, differing in detail from one adsorbate to another). Between descriptions of these two electronic investigations, we shall also present structural data for the adsorbed species on our chosen platinum surface.

### 3.1 Calculation of the Denominator for the Clean Surface

Here, we must evaluate the partial derivative
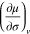
8that forms the denominator of eq 7. To do so, we have performed a series of calculations on slabs of various thicknesses within supercells of various lengths (for details, see Supporting Information). In each case, we separately impose positive and negative surface charges (of magnitude 0.02 e Å^–2^) which we denote σ^+^ and σ^–^. If the corresponding chemical potentials calculated via CASTEP are respectively denoted μ^+^ and μ^–^, then one might naively expect the desired partial derivative to be approximated by (μ^+^ – μ^–^)/(σ^+^ – σ^–^) but this would be to ignore the offset in chemical potentials due to the uniform artificial potential imposed by CASTEP in charged calculations. The effect of this offset does not reduce to zero as the vacuum separating neighboring slab images is increased, so the obtained partial derivative diverges with respect to vacuum thickness.

To overcome this problem, it will be necessary to correct the chemical potentials provided by CASTEP, ensuring that the constraint of constant external potential is properly imposed. We do so by first calculating the DOS for the positively and negatively charged systems, and aligning these so that their overlap is maximized in the region close to the bottom of the valence band (specifically, we maximize the cross-correlation of DOS over the energy region within 5 eV of the lowest valence eigenstate from each calculation; see Supporting Information). Since we expect modulation of the DOS caused by variation in the charge to primarily occur close to the Fermi level, it is reasonable to assert that shifts in the lower regions of the valence band are purely electrostatic in nature and arise wholly from the artificial potential imposed by CASTEP. With this alignment carried out, the raw chemical potentials provided by CASTEP are shifted to a common scale, and thus may sensibly be used (as suggested in the preceding paragraph) to obtain the denominator that we seek. The only ambiguity introduced by this procedure is that it is necessary to smooth the obtained DOS prior to alignment (even though we have used a rather dense sampling mesh) and the results do vary a little depending upon the smoothing used. We have applied Gaussian smoothing,^21^ with widths of either 0.1, 0.2, 0.3, or 0.4 eV, taking the average of the resulting denominator values as our nominal data points, while using the standard deviation as an error bar.

**Figure 1:**
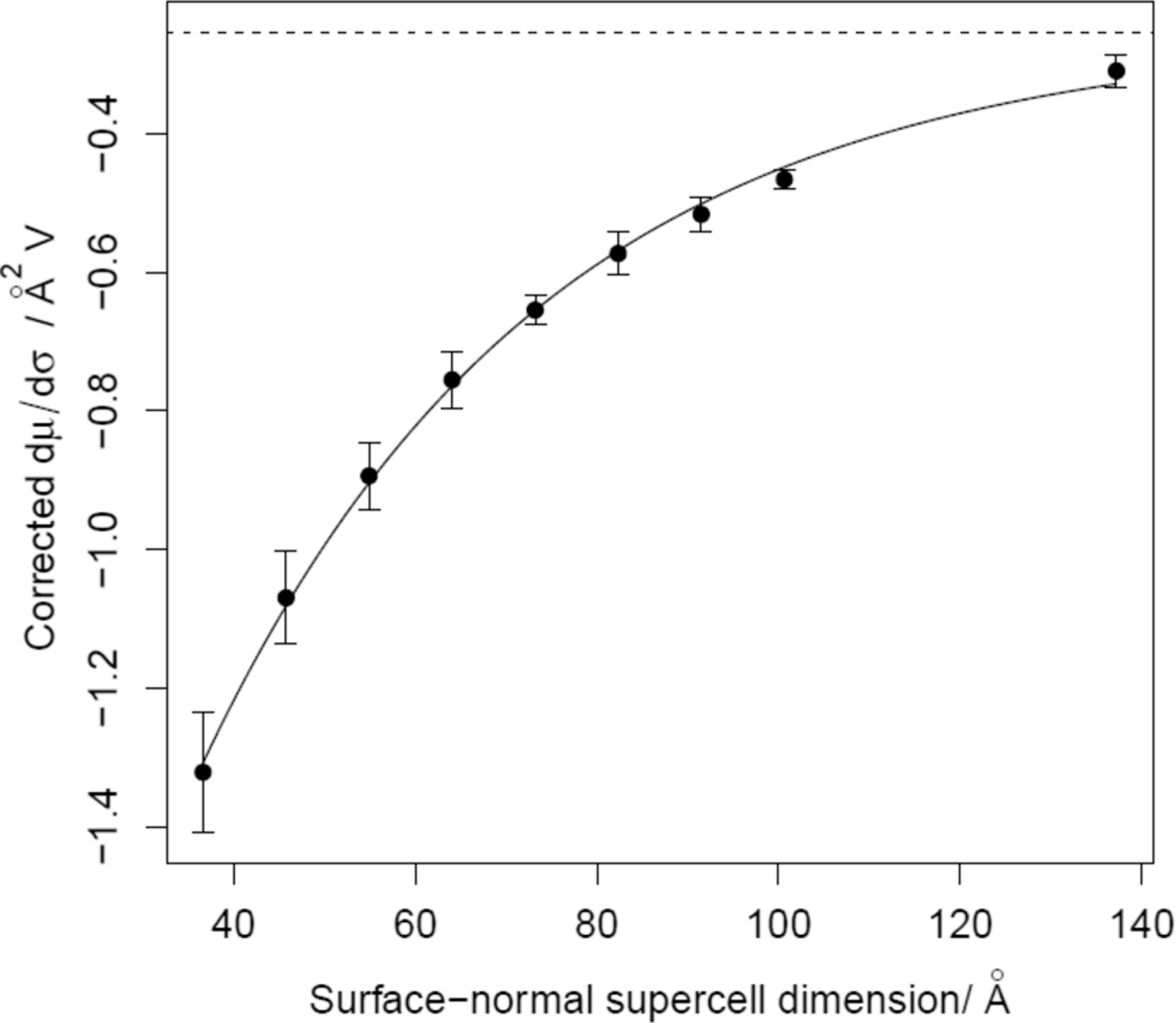
Denominator of local softness (eq 8) plotted as a function of surface-normal supercell dimension for clean Pt{111}. The dotted line indicates the asymptotic value (−0.254 Å^2^ V) based on an exponential fit.

In this way, we are able to avoid divergence in our computed values for the softness denominator, but unfortunately convergence remains conditional upon the manner in which we increase our system size. If we increase the slab thickness while keeping the vacuum thickness constant, we obtain a different result than if we had increased the vacuum thickness while keeping the slab thickness constant. It is thus impossible, we believe, to pin down a true value for the denominator (and hence for the local softness itself) that holds in all cases. Nevertheless, we believe that it is reasonable to impose, as a matter of definition, the condition that we shall treat as the “true” denominator that which is obtained by fixing the supercell length at twice the ideal thickness of the slab (so that the amount of vacuum is nominally identical to the amount of slab, notwithstanding the fact that the outermost layers of the slab are then allowed to relax). For example, in dealing with a slab comprising 12 layers we would employ a supercell of length sufficient to accommodate 24 layers at the bulk interlayer spacing. With this constraint imposed, we obtain the data displayed in Figure 1, which shows fairly smooth convergence toward a well-defined value for the denominator (−0.254 Å^2^ V). This is the value that we carry forward for use with the numerator derived in Calculation of the Numerator and Local Softness, believing it to be dominated by the metallic substrate contribution and not, therefore, requiring separate (expensive) recalculation with each new adsorbate. First, however, we must describe the adsorption geometries that we have calculated.

### 3.2 Optimized Geometries of Three Aromatic Adsorbates

Inspiration for the systems studied here was drawn from the work of Tan et al.,^16^ who reported details of anisole adsorption on Pt{111}. In that study, the molecule was bound to the surface through its aromatic ring, which was centered over one of the surface bridge sites and oriented such that two of its C–C bonds lay along a  in-plane direction. We investigated the same starting geometry, and used the same ring location and orientation for our studies of benzene and nitrobenzene also. In the benzene case, this also matches the preferred geometry found previously in studies from the groups of Neurock^23,24^ and Sautet.^25−28^ The side groups in nitrobenzene and anisole were placed so as to minimize steric repulsion between neighboring molecules within the (3 × 3) overlayer.

**Figure 2:**
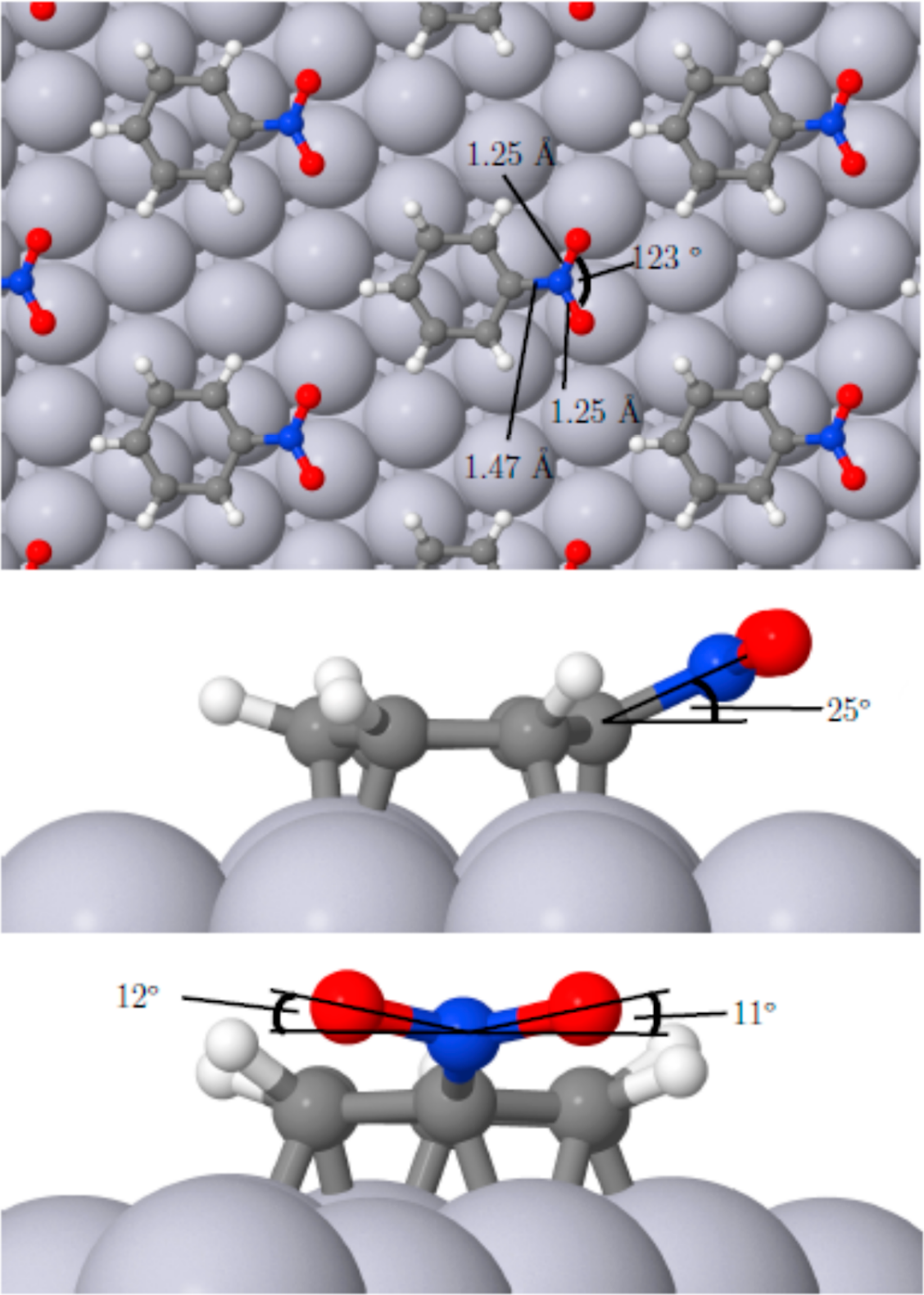
Top and side views of nitrobenzene adsorbed on Pt{111} in a (3 × 3) overlayer.

**Figure 3:**
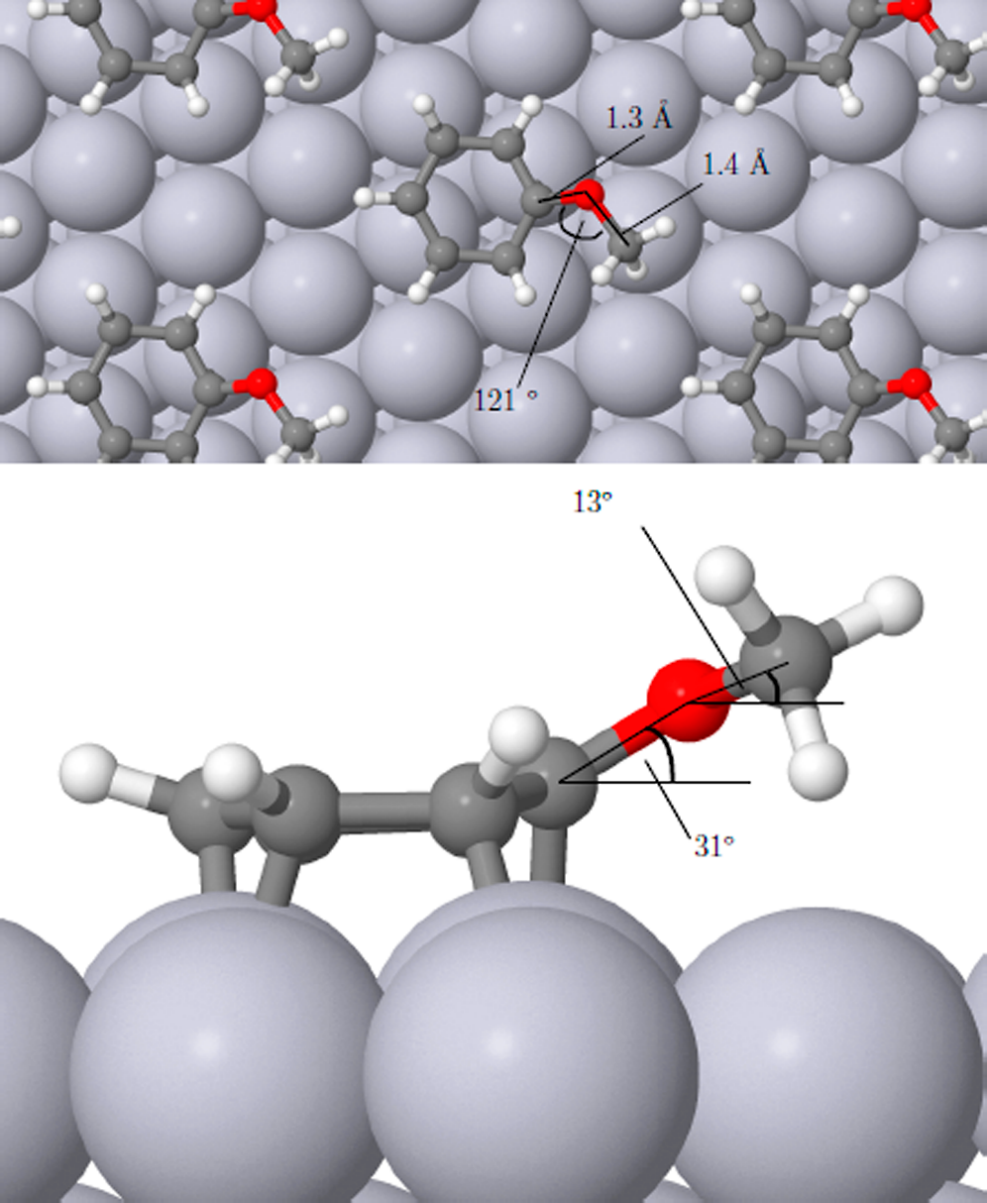
Top and side views of anisole adsorbed on Pt{111} in a (3 × 3) overlayer.

In all cases, the ring of the relaxed molecule remained essentially flat against the surface, with the side groups of nitrobenzene and anisole tilting slightly away (the C–N and N–O bonds in the former case lying 25° and 11/12° out of the surface plane, while the C–O bonds of the latter lay at 31° and 13°). Views of these adsorption geometries may be found in Figures 2 and 3. Our calculated adsorption heat for benzene was 0.99 eV per molecule, to be compared with 0.78 eV per molecule and 0.74 eV per molecule for anisole and nitrobenzene, respectively. Evidently the interaction between these two side groups and the surface is essentially repulsive in nature.

For ease of reference in the following subsection, it will be convenient to label the platinum atoms according to their bonding with carbon atoms (as per the convention of Yamagishi et al.^29^). In all three calculated adsorption geometries, we identify two platinum atoms per unit cell that bond to two carbon atoms each, and these we shall denote as type I. Similarly, there are two platinum atoms that bond to just one carbon atom each, which we shall describe as type II, and then a further five platinum atoms that bond to no carbon atoms, constituting type III. We can then, if we wish, note that each adsorbed ring contains four carbon atoms that bond only to type I platinum atoms, and two that bond only to type II platinum atoms, although we shall not confer any special name upon these.

### 3.3 Calculation of the Numerator and Local Softness

The numerator of eq 7
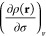
9was computed as the change in local electron density between calculations performed with surface charges of ±0.02 e Å^–2^ (with atomic positions frozen at the relaxed charge-free geometry). This was then divided by the clean-surface denominator computed as per Calculation of the Denominator for the Clean Surface, to obtain the local softness of the system. Our sign convention is such that a region with positive softness will see an increase in electron density if the chemical potential rises, or a decrease if the chemical potential falls. A region with negative softness, on the other hand, will see a decrease in electron density if the chemical potential rises, or an increase in electron density if the chemical potential falls.

**Figure 4:**
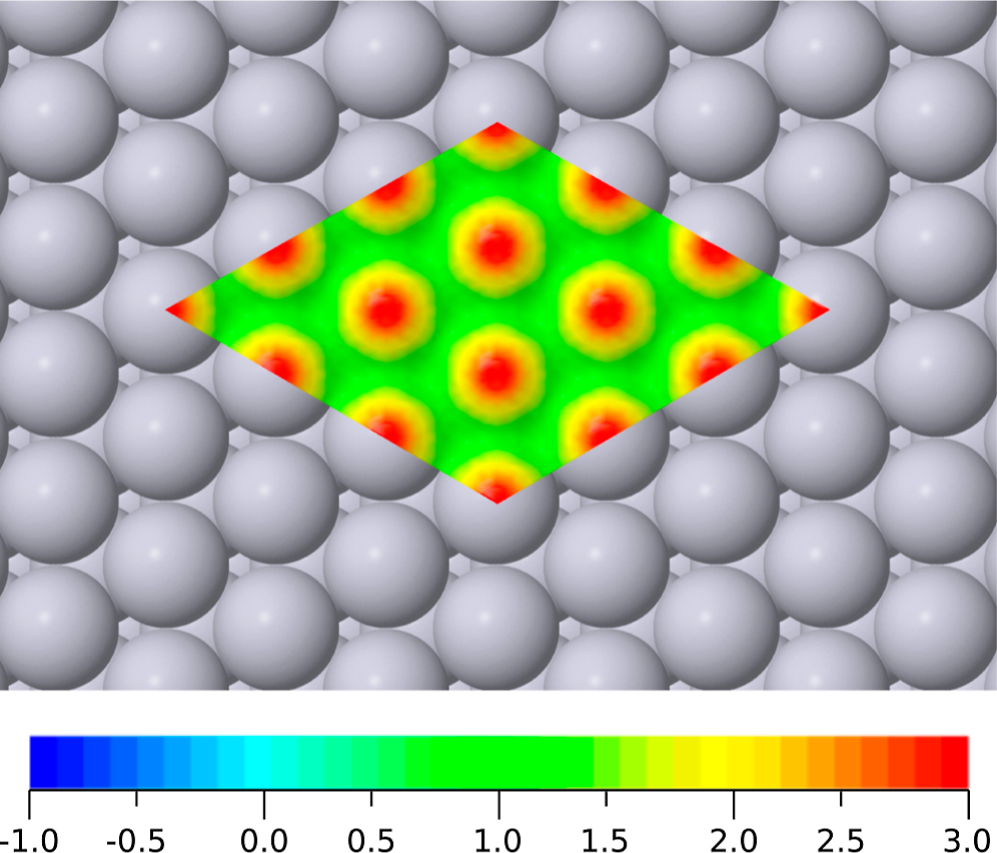
Top view of local softness at the clean Pt{111} surface, projected onto an isosurface where the valence electron density is one-fifth of its bulk value. The colormap ranges from −1 Å^–3^ V^–1^ (deep blue) to 3 Å^–3^ V^–1^ (deep red); the zero-point is cyan (not the midpoint of the color scale).

Our calculated local softness for the clean surface is conveniently presented (in Figure 4) projected onto an isosurface of constant valence electron density. Here, we see a region of strongly positive local softness (deep red) at the vertical extremity of each exposed atom, separated by much lower local softness (shades of yellow and green) in the vicinity of bridge and hollow sites. No regions of negative local softness (blue) are observed on the clean surface. A comparative study of local softness at {111}, {100} and {110} surfaces of multiple face-centered cubic metals will form part of a future publication, but for now we note that the qualitative picture is similar in each case.

**Figure 5:**
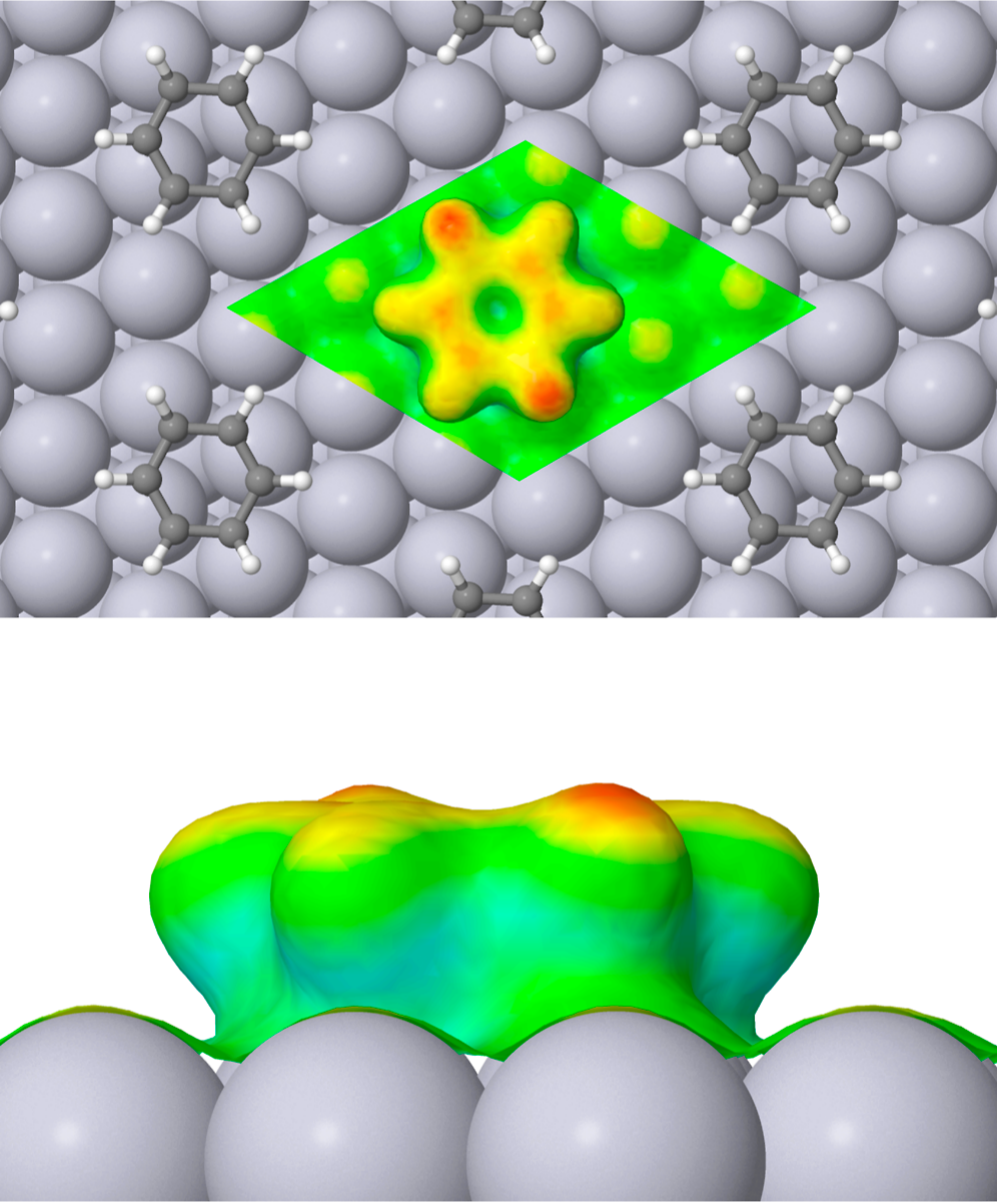
Top and side views of local softness for benzene adsorbed at the Pt{111} surface, details of presentation being identical to Figure 4.

In Figure 5, the local softness of adsorbed benzene is shown in the same manner. Here, we note that adsorption at this coverage is sufficient to suppress markedly the peak local softness (yellow instead of red) of even the type III platinum atoms, which we recall are not directly involved in binding to the molecule. There is a band of roughly zero softness between the molecule and the surface (cyan) but the upper region of the molecule is mostly of similar local softness to the maximum seen on the surface itself (yellow). Interestingly, however, there are peaks in the softness (red) on those carbon atoms that bond only with type I platinum atoms, but even more especially on the hydrogen atoms attached to those carbon atoms that bond only with type II platinum atoms. From the softness perspective, therefore, the molecule presents a distinctly 2-fold symmetry, reflecting the approximate symmetry of its binding site and orientation.

**Figure 6:**
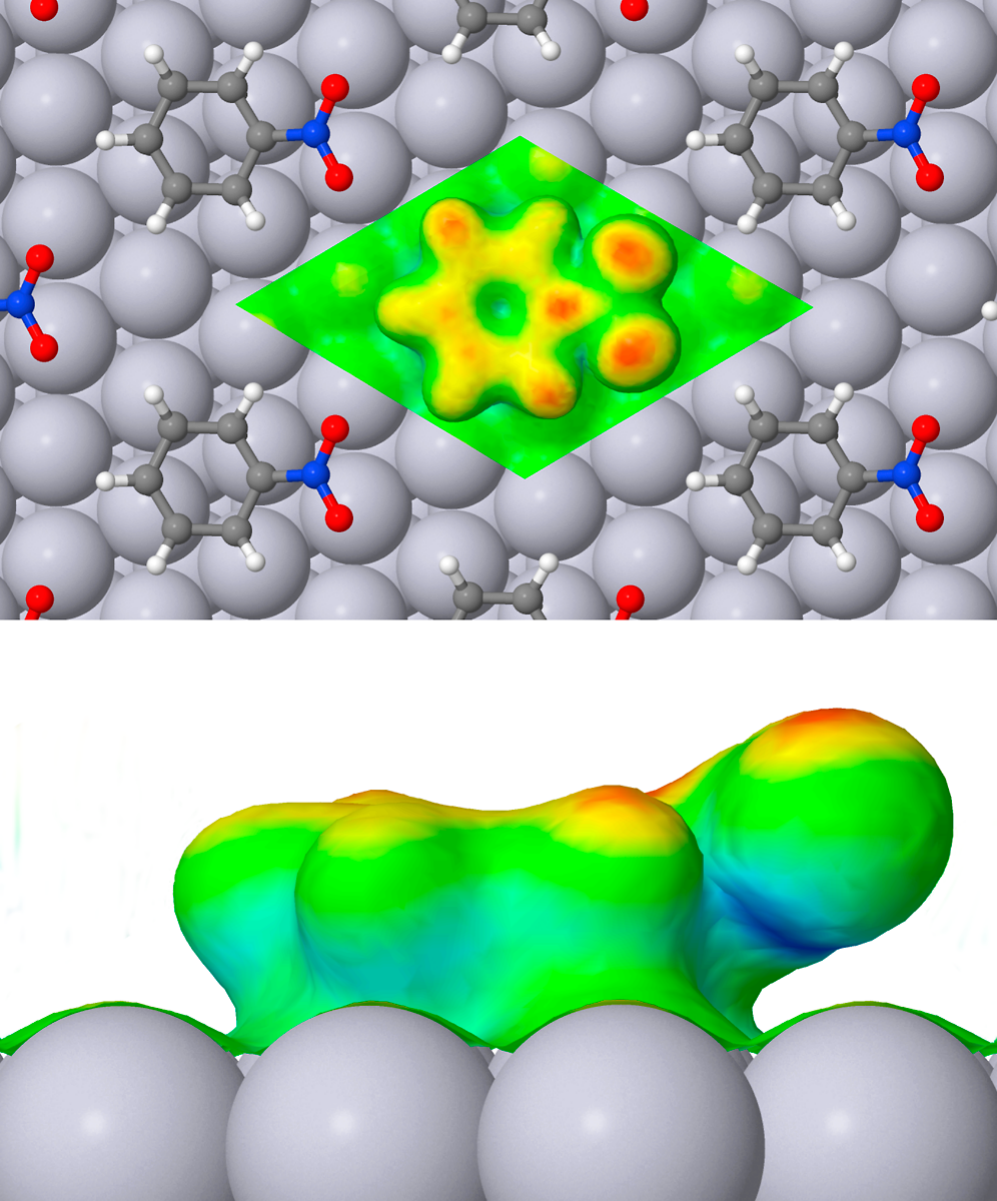
Top and side views of local softness for nitrobenzene adsorbed at the Pt{111} surface, details of presentation being identical to Figure 4.

Turning to the adsorption of nitrobenzene, its effect upon the softness of the type III platinum atoms (Figure 6) amounts to a rather similar degree of suppression (toward shades of yellow or green) to that observed upon the adsorption of benzene. Viewed from above, the distribution of high softness regions on the ring (red) is also similar to that seen with benzene, but there is also a quite distinctively high softness localized on that carbon atom to which the nitro group is attached. On the nitro group itself, the two oxygen atoms display high softness (red) while the nitrogen atom is apparently unremarkable from this perspective. Notably, however, the side view reveals a region of considerably negative softness (deep blue) located just beneath the nitrogen atom.

**Figure 7:**
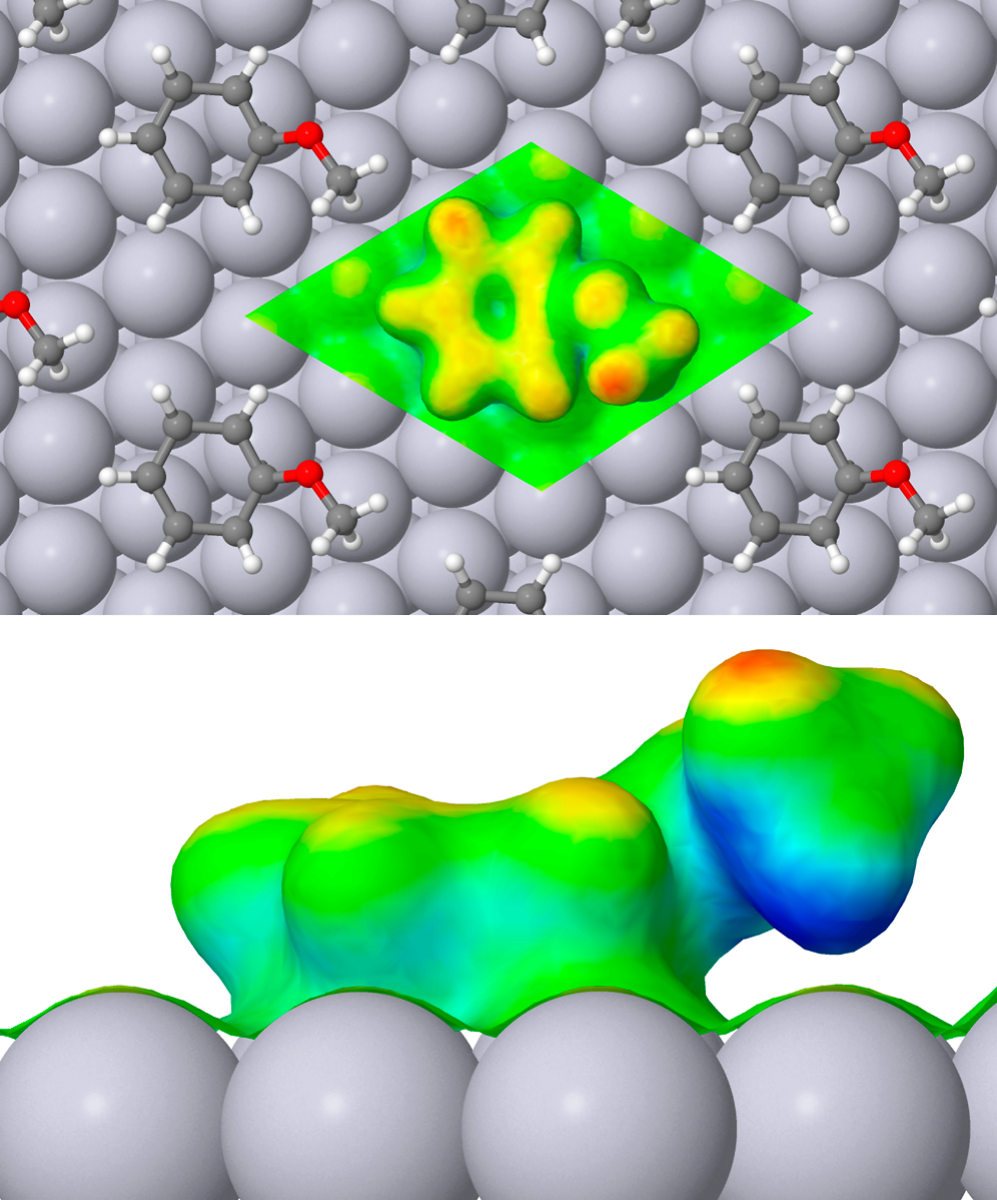
Top and side views of local softness for anisole adsorbed at the Pt{111} surface, details of presentation being identical to Figure 4.

Finally, as far as the local softness is concerned, the adsorption of anisole (Figure 7) again suppresses that of the type III platinum atoms (no more than yellow) but now the distribution of higher softness (red) on the ring is less obvious than before. The carbon atom to which the side group attaches is no longer a softness hotspot (remaining green) and the oxygen atom too is only modestly soft (essentially yellow). In this case, the locally softest part of the molecule is undoubtedly the methyl hydrogen atom that lies closest to the ring (red) while the methyl hydrogen atom that points down toward the surface harbors by far the most negative local softness of the molecule, along with the underside of the methyl carbon atom (both deep blue). The third methyl hydrogen atom is of intermediate local softness (yellow).

**Figure 8:**
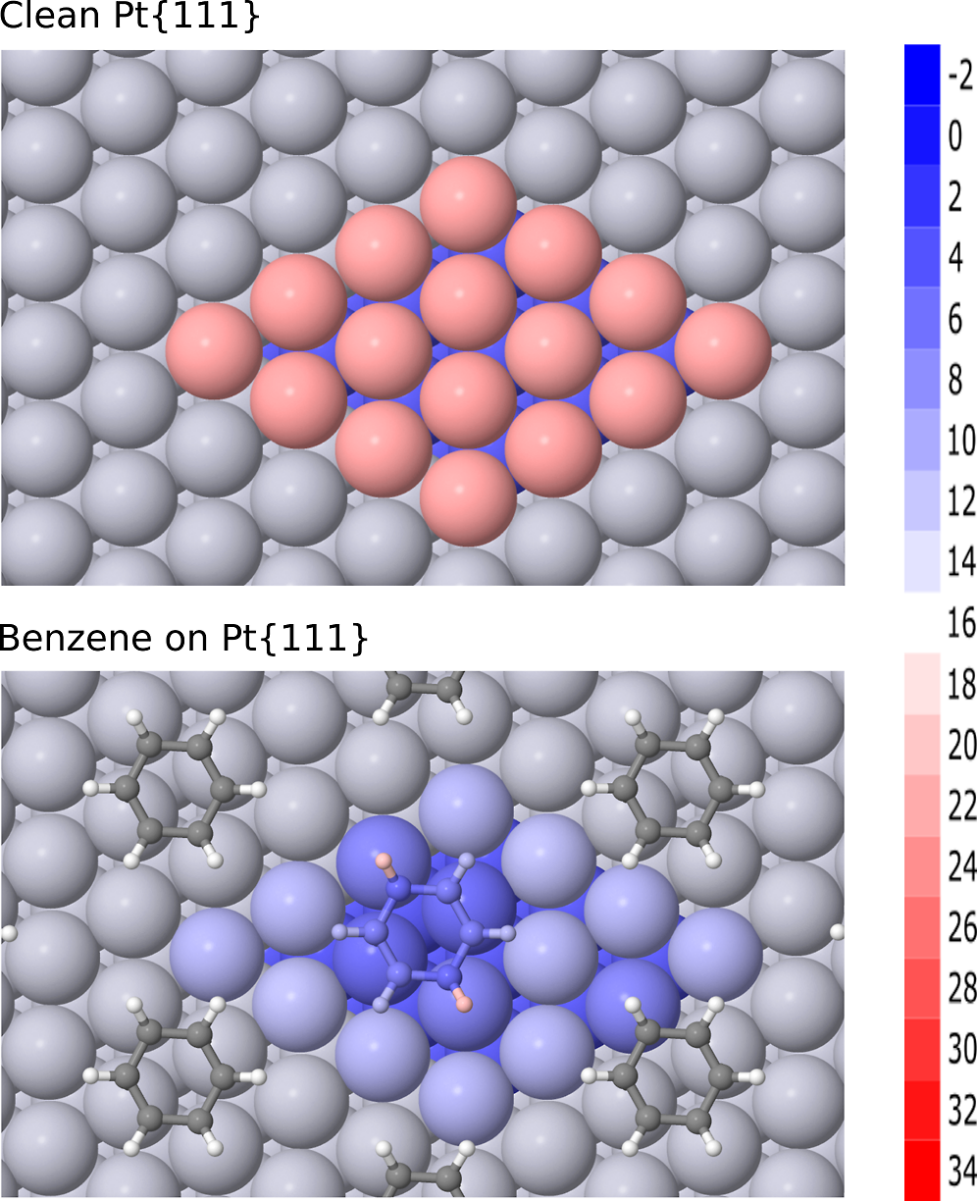
Top views of atomic softness for the clean Pt{111} surface (upper panel) and for benzene adsorbed on the same (lower panel). The colormap ranges from −2 eV^–1^ (deep blue) to 34 eV^–1^ (deep red).

Returning to the clean surface, we now display the atomic softness (i.e., the integral of local softness over atomic volumes determined via the topological method) in Figure 8 (upper panel). Here, the strongly positive atomic softness of the uppermost layer (pink) can clearly be seen to contrast with the much less strongly positive atomic softness of the second and third layers (blue). Such information is typically obscured when displaying the local softness in the manner of Figure 4, suggesting that the two approaches to data presentation are usefully complementary.

In Figure 8 (lower panel) we show the atomic softness for adsorbed benzene, and again see more detail for the platinum atoms than was visible in the previous plot of local softness (Figure 5). Specifically, it becomes clear that the type I platinum atoms are the least soft within the uppermost layer, followed by the type II and finally the type III platinum atoms (varying shades of blue). As for the adsorbate itself, the four carbon atoms bonding to type I platinum atoms appear very slightly softer than the two carbon atoms bonding to type II platinum atoms, but by far the most prominent feature is that the two hydrogen atoms attached to the latter carbon atoms are quite distinctly the softest of the entire system (light pink). These observations are entirely consistent with the local softness picture describe above, but the discrete nature of the image arguably renders the information more clearly.

**Figure 9:**
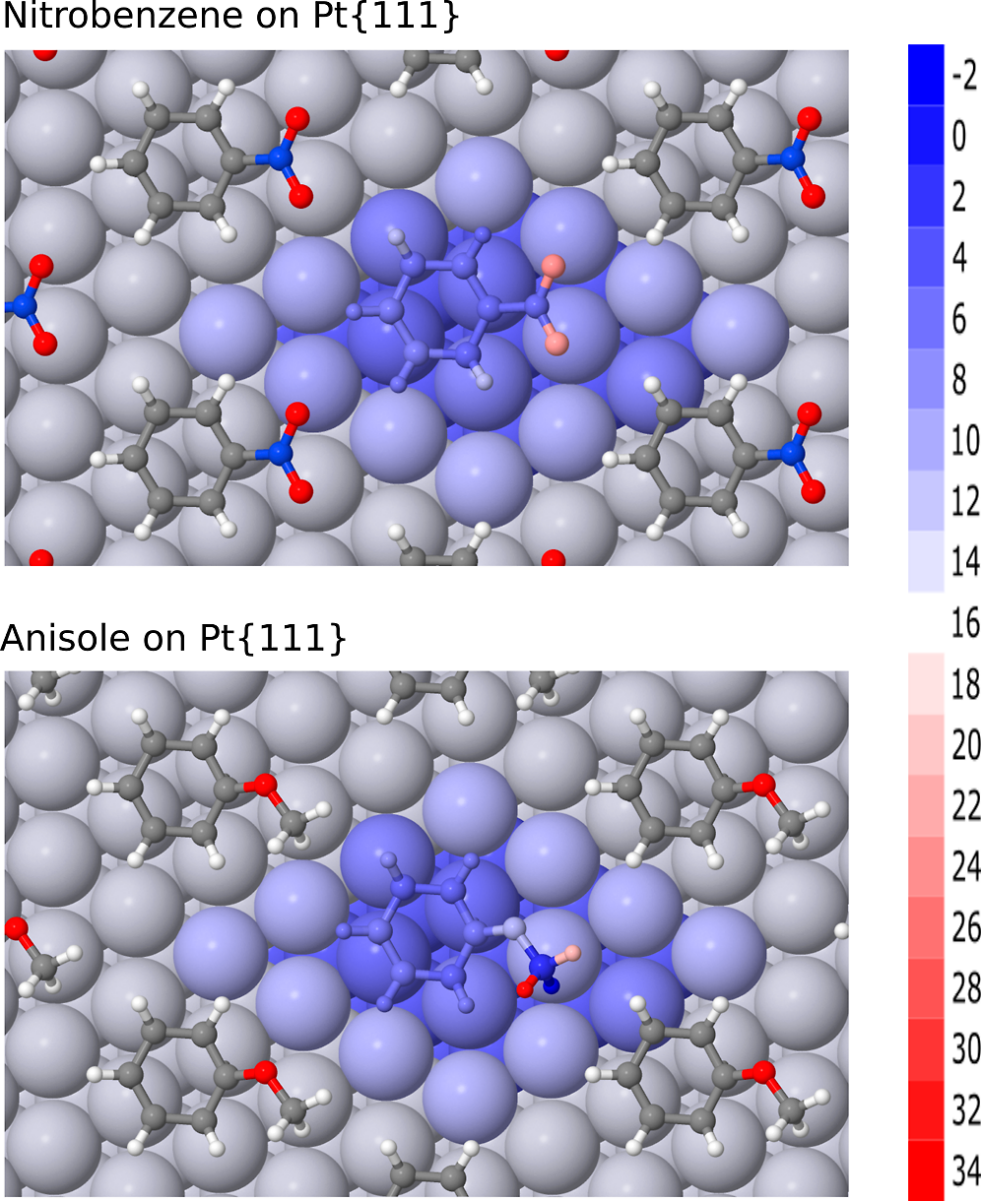
Top views of atomic softness for nitrobenzene adsorbed on the Pt{111} surface (upper panel) and for anisole adsorbed on the same (lower panel). The colormap ranges from −2 eV^–1^ (deep blue) to 34 eV^–1^ (deep red).

A similar situation may be sketched for adsorbed nitrobenzene, shown in Figure 9 (upper panel). As in the case of benzene, the least soft atoms of the uppermost layer are the type I platinum atoms, followed by the type II and type III platinum atoms in order (shades of blue). The carbon atoms show barely perceptible differences in softness (all mid blue) even for the atom that binds to the nitro group, implying that some detail from the local softness plot (Figure 6) is lost when integrating over topologically defined atomic envelopes, but two of the hydrogen atoms do show distinctly higher softness (light blue) in the same positions as for benzene. The softness of the nitrogen atom is similar to that of the carbon atoms (mid blue) while that of the two oxygen atoms is the highest of all (pink).

Turning back to adsorbed anisole, Figure 9 (lower panel) reveals much the same pattern of atomic softness within the uppermost platinum layer as for the other adsorbates, so we will not describe it again. As regards the adsorbate itself, there is now very little to distinguish between the carbon and hydrogen atoms of the ring (similar shades of blue) in line with our observation based on local softness (see discussion of Figure 7 above). The oxygen atom, by contrast, is noticeably softer than the ring atoms (light blue) while the methyl carbon atom actually shows negative softness (deep blue). Of the three methyl hydrogen atoms, the one lying closest to the ring shows strong positive softness (deep red), while the downward-pointing example shows negative softness (deep blue) and the third is intermediate (light pink). Again, this is consistent with the corresponding local softness image (Figure 7) but arguably more distinct.

## 4 Conclusions

Our present results illustrate the utility of local softness in identifying regions within adsorbed molecules that are susceptible to chemical reaction. These are the regions where electrons will either (a) most readily accumulate upon reduction, or (b) most readily be withdrawn upon oxidation. In the case of benzene adsorption, we have shown that the distribution of local softness reflects the 2-fold symmetry of its preferred adsorption site, being concentrated predominantly on two of the molecule’s hydrogen atoms and four of its carbon atoms. The same pattern is essentially replicated for the ring of nitrobenzene, except that the carbon atom binding the nitro group is especially soft; within the substituent itself, the two oxygen atoms are the softest atoms of the entire molecule, while the region beneath the nitrogen atom is particularly hard. For anisole, the softness within the ring again reflects the site geometry, albeit with variations that are less pronounced, and the oxygen atom is once more somewhat soft; most notably, however, the methyl carbon atom is distinctly hard, while its three hydrogen atoms range from hard to extremely soft.

The striking feature common to the systems is that little evidence of directing effects survives contact between the substituted molecules and the surface. We might have expected softness to correlate with *ortho*, *meta*, or *para* sites, but instead the bonding to platinum dominates the electronic structure of the ring in each case. In fact, this is consistent with the conclusions reached by Tan et al. for anisole,^16^ based upon inspection of electron density difference plots in comparison with molecular orbitals of the free molecule. A quantitative expression of similar ideas has also been presented by Réocreux et al., resting upon the hard and soft acids and bases concept (HSAB).^30^ In essence, out-of-plane deformation of the adsorbed molecule compromises conjugation between the aromatic moiety and the methoxy group, rendering the latter less effective in its role as an electron-donating substituent. Similarly, out-of-plane deformation in nitrobenzene also appears to compromise the electron-withdrawing effect of its substituent, for much the same reason. These observations underline the propensity of adsorption systems to confound our expectations, and the necessity of supplementing our understanding with calculated descriptors of electronic structure and reactivity.

## Data Availability

The data underlying this study are openly available in the University of Cambridge Data Repository (Apollo) at 10.17863/CAM.109686.

## Supporting Information Available

It contains details of the supercells and cross-correlation procedure referred to in Calculation of the Denominator for the Clean Surface (PDF)
