# Supplementary material for: Chemical Softness in Aromatic Adsorption: Benzene, Nitrobenzene and Anisole on Pt{111}
Source: J Phys Chem A. 2024 Jul 22;128(30):6296–304. doi: 10.1021/acs.jpca.4c02214 (PMC11299172; doi:10.1021/acs.jpca.4c02214)
Supplement: Supplementary file 1 — jp4c02214_si_001.pdf [file jp4c02214_si_001.pdf]

Chemical Softness in Aromatic Adsorption: Benzene, Nitrobenzene and Anisole on Pt{111}

Amy L. Gunton
Department of Physics and Astronomy,
University of Exeter, Stocker Road,
Exeter, EX4 4QL, United Kingdom

Stephen J. Jenkins
Yusuf Hamied Department of Chemistry, University of Cambridge,
Lensfield Road, Cambridge CB2 1EW, United Kingdom

(Dated: June 26, 2024)

SUPERCELL DIMENSIONS

Supercell dimensions were consistent with a platinum lattice constant of 3.961 Å, computed in separate bulk calculations with compatible convergence parameters. For the (1×1) cells used in calculating the softness denominator, the surface unit cell was therefore spanned by two vectors of length 2.801 Å (at 60° separation) while for the (3×3) cells used in calculating the numerator these vectors were 8.403 Å in length.

In the denominator calculations, various slab thicknesses and supercell lengths were used, as listed in Table S1. In the numerator calculations, the supercell length was 25.156 Å – equivalent to 11 ideal {111} layers at the calculated platinum lattice constant.

TABLE S1. Supercell lengths used in evaluating the softness denominator, expressed both as an integer number of {111} layers and in Ångstrom units. In each case, the supercell contained a slab comprising half the total number of layers, with the remainder left empty. For example, the 28-layer supercell contained a 14-layer slab, and so on.

Length (Layers)	Length (Å)
16	36.591
20	45.739
24	54.886
28	64.034
32	73.182
36	82.329
40	91.477
44	100.625
60	137.216

DETAILS OF CROSS-CORRELATION METHOD

As discussed briefly in the main text, we have aligned the energy bands arising from positively and negatively charged calculations with one another, to compensate for changes in the artificial background potential imposed by CASTEP in these cases. This we have done by means of a cross-correlation procedure, described in more detail here.

Given two density of state functions, $g_1(\epsilon)$ and $g_2(\epsilon)$, their cross-correlation may be defined as

$$f(\tau) = \int_{-\infty}^{\infty} g_1(\epsilon) g_2(\epsilon + \tau) d\epsilon \tag{1}$$

which will take its maximum value for the value of the offset variable, τ, that renders the two functions most similar. Having determined this value of τ, we may simply apply such an offset to the chemical potential corresponding to $g_2(\epsilon)$ before evaluating Eqn. 8 (from the main paper).

In our case, however, we wish to align just the lowest-energy portion of the density of states curves, where we expect the effect of charge modulation to give rise only to a rigid electrostatic shift. Close to the Fermi level, the density of states profile changes non-rigidly, compromising the usefulness of the cross-correlation method. From a practical perspective, limiting the cross-correlation comparison to the lower portion of the density of states curves was achieved by multiplying $g_1(\epsilon)$ and $g_2(\epsilon)$ by a Fermi-Dirac function whose effective chemical potential was set to 5 eV above the lowest energy eigenvalue reported by CASTEP in each case (the valence bands extending to around 10 eV below the Fermi level). The results are hardly sensitive to reasonable choices of the distribution width, and in our case this was chosen somewhat arbitrarily such that $k_B T = 0.4545$ eV. This corresponds to an effective temperature of 5275 K, but no particular physical meaning attaches to that number. A rather sharper or smoother cutoff could be applied without unduly affecting the result.
